# Oral HPV infection and MHC class II deficiency (A study of two cases with atypical outcome)

**DOI:** 10.1186/1476-7961-10-6

**Published:** 2012-04-23

**Authors:** Naouel Guirat-Dhouib, Yemen Baccar, Imène Ben Mustapha, Monia ouederni, Sameh Chouaibi, Nadia El Fekih, Mohamed Ridha Barbouche, Bassima Fezaa, Ridha Kouki, Slama Hmida, Fethi Mellouli, Mohamed Bejaoui

**Affiliations:** 1 Centre National de Greffe de Moelle Osseuse, Service d'immuno-Hématologie Pédiatrique, Faculté de Médecine de Tunis 2, Rue Djebel Lakhdhar, 1006 Tunis, Tunisia; 2 Pasteur 13, Place Pasteur, B.P. 74., 1002 Tunis, Belvédère, Tunisia; 3 Hôpital Charles Nicolle, Boulevard 9 Avril 1938, Tunis 1006, Tunisia; 4 Centre national de transfusion sanguine 13, Rue Djebel Lakhdhar, Tunis, Tunisia

## Abstract

**Background:**

Major histocompatibility complex class II deficiency, also referred to as bare lymphocyte syndrome is a rare primary Immunodeficiency disorder characterized by a profondly deficient human leukocyte antigen class II expression and a lack of cellular and humoral immune responses to foreign antigens. Clinical manifestations include extreme susceptibility to viral, bacterial, and fungal infections. The infections begin in the first year of life and involve usually the respiratory system and the gastrointestinal tract. Severe malabsorption with failure to thrive ensues, often leading to death in early childhood. Bone marrow transplantation is the curative treatment.

**Case reports:**

Here we report two cases with a late outcome MHC class II deficiency. They had a long term history of recurrent bronchopulmonary and gastrointestinal infections. Bone marrow transplantation could not be performed because no compatible donor had been identified. At the age of 12 years, they developed oral papillomatous lesions related to HPV (human papillomavirus). The diagnosis of HPV infection was done by histological examination. HPV typing performed on the tissue obtained at biopsy showed HPV type 6. The lesions were partially removed after two months of laser treatment.

**Conclusions:**

Viral infections are common in patients with MHC class II and remain the main cause of death. Besides warts caused by HPV infection do not exhibit a propensity for malignant transformation; they can cause great psychosocial morbidity.

## Case presentations

### Case 1

**Figure 1:**
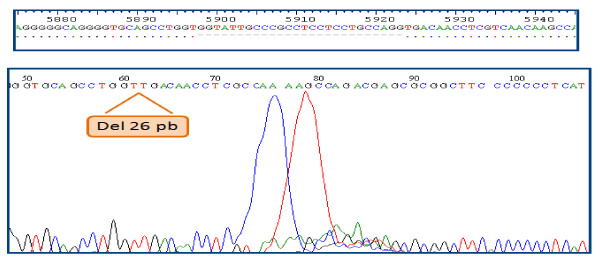
***RFXANK *mutation**.

**Figure 2:**
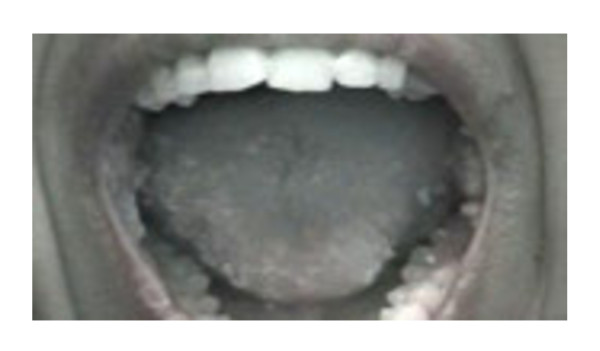
**Disseminated warts on oral cavity**.

A 22-year-old girl, born from unrelated healthy parents of Tunisian origin, during the neonatal period had received BCG immunization without any local or systemic responses. From 6 months of age she had recurrent episodes of bronchopulmonary and gastrointestinal infections. At 10 years, she was referred to our hospital for the first time. On physical examination, she was growth retarded with a weight of 20 kg (-2.2SD) and height of 121 cm (-2SD). Tuberculin skin test was negative. Immunologic examination revealed decreased serum IgG levels: 5 g/l (5.6-16.1), low serum IgA: 0.4 g/l (0.5-2.5), and serum IgM levels within the normal range of age: 0.8 g/l (0.5-1.8). Lymphocyte subsets produced the following: CD_3 _= 2 × 10^9^cells/L (1.2-2.6), CD_4 _= 1 × 10^9^cells/L (0.65-1.5), CD_8 _= 1 × 10^9^cells/L (0.37-1.1), CD_19 _= 0.374 × 10^9^cells/L (0.27-0.86). The diagnosis of MHC class II deficiency was established on the basis of the loss of MHC II expression on peripheral blood lymphocytes and PHA activated T cells related to homozygous *752delG25 *mutation in the *RFXANK *gene (Figure 1). She was started on intravenous immunoglobulin (0.4 g/kg by 3 weeks) and cotrimoxazole sulfamethoxazole prophylaxis (25 mg/kg, 3 times a week). Bone marrow transplantation could not be performed because no compatible donor was identified. At the age of 12, she had multiple, pale, painless, lesions on her buccal mucosa (Figure 2). The lesions were firm on palpation, covered by healthy, normal appearing mucosa, neither ulcerated nor inflamed, but her dental hygiene was poor. Sexual abuse was denied by the patient. A biopsy of the oral lesions with histological examination was performed; HPV infection was diagnosed. HPV typing performed on the tissue obtained at biopsy showed HPV type 6. HLA typing revealed an HLA DQB1*0301phenotype. The patient was treated by laser with partial response. She has a Karnofsky Performance Scale Index score of 50%.

### Case 2

**Figure 3:**
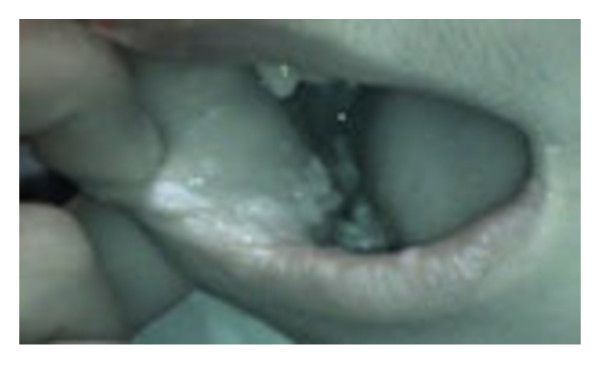
**Inferior intern lip (right side)**.

A 19-year-old girl, the sister of the index case, had had an uneventful clinical course from birth since the sixth month of life when she has suffered from chronic diarrhea and recurrent bronchopulmonary infections with bronchiectasis. Immunologic examination revealed decreased serum IgG levels: 4.5 g/l (5.6-16.1), low serum IgA: 0.46 g/l (0.5-2.5), and serum IgM levels within the normal range for age: 0.8 g/l (0.5-1.8). Lymphocytes subsets produced the following: CD_3 _= 2.7 × 10^9^cells/L (1.2-2.6), CD_4 _= 1.4 × 10^9^cells/L (0.65-1.5), CD_8 _= 1.65 × 10^9^cells/L (0.37-1.1), CD_19 _= 0.46 × 10^9^cells/L (0.27-0.86). The diagnosis of MHC class II deficiency was based on the family and personal history of recurrent and severe infections, and was confirmed at six years of life when she was referred to our hospital. Immunologic examination revealed a defect of HLA-DR expression comparable to that in her sister. The molecular analysis identified the same homozygous 752delG26 mutation in the RFXANK genes. Intravenous immunoglobulin (0.4 g/kg every 3 weeks) and cotrimoxazole sulfamethoxazole prophylaxis (25 mg/kg, 3 times a week) were initiated after this diagnosis. Bone marrow transplantation could not be performed because no compatible donor had been identified. Her symptoms improved between the ages of 5 and 12 years, with a decrease in the frequency and severity of infections. At the age of 12, exophytic and cauliflower papillomatous lesions appeared with clear contours at labial and buccal mucous membrane, without other inflammatory lesions (Figure 3). These lesions were not associated with dysphagia, odynophagia or respiratory problems; HPV typing performed on the tissue obtained at biopsy showed HPV type 6. HLA typing revealed an HLA DQB1*0301phenotype. The lesions were partially removed after two months of laser treatment. She has a Karnofsky Performance Scale Index score of 50%.

## Discussion

Lymphocyte major histocompatibility complex (MHC) class II deficiency also referred to as the bare lymphocyte syndrome, is a rare autosomal recessive primary combined immunodeficiency syndrome that accounts for 5% of all cases of severe combined immunodeficiency and characterized by a defective expression of human leucocyte antigen (HLA) class II molecules due to mutations in four different MHC II regulatory genes (CIITA, RFXANK, RFX5, RFXAP) [1]. More than 150 patients have been reported worldwide, most of them were originated from North Africa where the frequency of consanguineous marriages is high [2,3]. Half of the reported cases have *RFXANK *deficiency [2-4]. Clinical manifestations appear early in life including susceptibility to infections, primarily of the respiratory and gastrointestinal tract and severe malabsorption with failure to thrive often leading to death in early childhood [5,6]. Supportive treatment includes intravenous immunoglobulin and prophylaxis against *Pneumocystis Jovecci*. This therapy results in a marked decrease in the number of bacterial infectious episodes. MHC class II deficiency has a poor prognosis, with a life expectancy of only a few years [7]. The only curative approach known to date is allogeneic haematopoietic stem cell transplantation [8]. Few patients survive beyond the age of 20 years under regular immunoglobulin substitution and prophylactic antibiotics. Unknown genetic factors may influence innate or CD8-T cell-mediated immunity, potentially accounting for this more favorable outcome. Genetic predisposition factors and environmental factors such as medical care and family hygiene may be responsible for differences in the clinical expression of the disease state [9]. Affected patients are extremely susceptible to persistent and recurrent viral infections. The most frequent viral infections include Cytomegalovirus, enterovirus, adenovirus and herpes simplex virus [4]. MHC class II deficiency patients are at increased risk of oral HPV infection. Negative tuberculin skin test, lymphopenia, and the low CD4+ T cell count indicated that our patients had defects in cellular immunity, which lead to the HPV infection. Genetic factors, malnutrition, and poor hygiene are also found to be associated with this infection. Accompanying features such as growth retardation and poor oral hygiene of our patients might have been facilitating factors. According to other studies [10,11], the major histocompatibility complex might contribute to the HPV infection, however HLA typing of our patients revealed an HLA DQB1*0301phenotype. HPV infection has attracted a great deal of attention, not just because of the difficulty of managing oral warts but also because of the oncogenic potential of certain strains [12]. HPV has been implicated as a cause of several types of benign oral lesions grouped clinically as oral warts. Oral warts can present in almost any location in the mouth as nodular or raised lesions that appear pink or white depending on the degree of keratinization. The majority of these lesions are the result of HPV 6 and 11 [13]. HPV types 6 and 11 are typically labeled as low risk because infection with these types has low oncogenic potential and usually results in the formation of condylomata and low-grade precancerous lesions. Several treatment options are used to remove oral warts including electro or radiodissecation, laser surgery, cryotherapy, or surgical excision but no treatment is completely satisfactory; relapse is frequent and requires re-treatment [14]. The lesions of our patients were partially removed by laser surgery.

## Conclusions

Viral infections are commonly reported in patients with combined immunodeficiency. These reports add to the list of serious infections occurring in association with MHC class II deficiency. Allogeneic hematopoietic stem cell transplantation is the only therapeutic option available for patients with MHC class II deficiency.

## Competing interests

The author declares that they have no competing interests.

## Authors' contributions

NGD-organized, contributed to manuscript preparation, generated all figures and developed the format;YB organized manuscript; IBM-contributed to molecular and immunological evaluation of these case reports; MO-assisted with manuscript review references; SC-assisted in writing treatment portion; NEF-performed skin biopsies; MRB- contributed to molecular and immunological evaluation of these case reports; BF-performed skin biopsies; RK-organized manuscript; SH-conducted HLA typing; FM-contributed to literature search; MB-assisted with critically revised the manuscript and corrections. All authors read and approved the final manuscript.
